# Regulation of ATP production by mitochondrial Ca^2+^

**DOI:** 10.1016/j.ceca.2012.03.003

**Published:** 2012-07

**Authors:** Andrei I. Tarasov, Elinor J. Griffiths, Guy A. Rutter

**Affiliations:** a Section of Cell Biology, Division of Diabetes Endocrinology and Metabolism, Department of Medicine, Imperial College London, SW7 2AZ, London, UK; b Department of Biochemistry, School of Medical Sciences, and Bristol Heart Institute, University of Bristol, Bristol BS8 1TD, UK

**Keywords:** [ATP/ADP]_c_, cytosolic ATP/ADP ratio, [Ca^2+^]_c_, [Ca^2+^]_m_, cytosolic and mitochondrial free concentrations of calcium, EC, excitation-contraction, ICEU, intracellular energy unit, K_ATP_, ATP-sensitive K^+^ channel, MCU, mitochondrial uniporter, mNCX/NCLX, mitochondrial sodium-calcium exchanger, Pi, PPi, inorganic phosphate and pyrophosphate, respectively, RuR, ruthenium red, ATP, Mitochondria, Calcium, Heart, Beta cell, Islet, Insulin, Secretion, Diabetes

## Abstract

Stimulation of mitochondrial oxidative metabolism by Ca^2+^ is now generally recognised as important for the control of cellular ATP homeostasis. Here, we review the mechanisms through which Ca^2+^ regulates mitochondrial ATP synthesis. We focus on cardiac myocytes and pancreatic β-cells, where tight control of this process is likely to play an important role in the response to rapid changes in workload and to nutrient stimulation, respectively. We also describe a novel approach for imaging the Ca^2+^-dependent regulation of ATP levels dynamically in single cells.

## 1 Introduction

Mitochondria are the major site of oxidative metabolism, and hence ATP synthesis, in eukaryotic cells. Thus, whereas the glycolytic metabolism of glucose generates 2 ATP molecules, 36–38 are generated by the reactions of the citrate cycle and the oxidation of the resulting NADH and FADH_2_ by the respiratory chain [1]. The latter reactions are tightly controlled by intramitochondrial Ca^2+^ in a mechanism designed to ensure that ATP synthesis is closely coupled to the cell's energetic needs. We discuss this form of regulation in detail here, taking as examples two cell types with contrasting energetic needs, the cardiac myocyte and the pancreatic β cell.

## 2 Ca^2+^ regulation of intramitochondrial oxidative metabolism: a brief historical perspective

Mitochondrial oxidative metabolism has long been recognised as subject to complex regulation by several factors, notably the concentrations of ADP and substrate(s) [2]. However, regulation by Ca^2+^ has emerged in recent years as a further important means of controlling this vital aspect of cell function. Mammalian mitochondria have a huge capacity for Ca^2+^ uptake and for many years it was thought that these organelles served as mobilisable intracellular reservoirs of Ca^2+^ ions [3]. Studies in the 1970s and 80s by Denton and McCormack [4] as well as Hansford and Castro [5] and Crompton [6] indicated instead that under basal conditions total mitochondrial Ca^2+^ content was low and that increases in cytosolic free Ca^2+^ ([Ca^2+^]_c_) in response to extrinsic agents (nutrients, hormones, neurotransmitters, etc.), were likely to provoke increases in intramitochondrial free Ca^2+^ ([Ca^2+^]_m_) concentrations. Thus, based on the properties of a group of key intramitochondrial dehydrogenases (later characterised in some detail – see the following sections) [7,8], it was proposed that the uptake of Ca^2+^ ions from the cytosol increased [Ca^2+^]_m_ from ∼0.1 to 10 μM or more. The consequent activation of oxidative metabolism would then provide an increased supply of reducing equivalents to drive respiratory chain activity and ATP synthesis. Export of mitochondrial ATP in exchange for ADP was anticipated then to meet an increased ATP demand to fuel energy-requiring processes in the cytosol such as ion pumping, contraction, exocytosis etc. The biochemical analyses (e.g. [9]) upon which the above model was based were subsequently complemented and reinforced in the 1990s by highly selective measurements of intramitochondrial free Ca^2+^ in living cells by Rizzuto, Pozzan, Rutter and others [10,11]. These experiments used recombinant expression of the Ca^2+^-sensitive photoprotein aequorin, targeted to the mitochondrial matrix by in-frame fusion with the signal peptide of cytochrome c subunit VIII. Later extended by Rutter et al. to measurements at the level of single cells [12], this approach provided evidence that basal intramitochondrial Ca^2+^ levels were indeed low (similar to or lower than those in the cytosol, i.e. ≤100 nM) and that the free concentrations of the ion increased within mitochondria in response to Ca^2+^-mobilising agonists. Subsequent refinements of these approaches, including the use of GFP-based probes, highlighted the importance of close contacts between the endoplasmic reticulum and mitochondria in the uptake of Ca^2+^ by the latter [13] and demonstrated that mitochondrial Ca^2+^ increases stably elevate mitochondrial [ATP] [14].

## 3 Mitochondrial Ca^2+^ transport: roles of the mitochondrial uniporter MCU and the Na^+^/Ca^2+^ exchanger, mNCX/NCLX

An impediment to progress in this field has been that the mitochondrial Ca^2+^ transport proteins were not purified or cloned, and that use of the inhibitors of these pathways is problematic in living cells. However, recent work has very likely identified the proteins responsible for both the Ca^2+^ influx and Ca^2+^ efflux pathways in mitochondria (see below).

Increases in [Ca^2+^]_c_ are relayed to the mitochondria by the mitochondrial Ca^2+^ uniporter (MCU), resulting in activation of mitochondrial dehydrogenases and stimulation of ATP synthesis. Average [Ca^2+^]_m_ is controlled by the activities of the MCU and the efflux pathway, the mitochondrial Na^+^/Ca^2+^ exchanger (mNCX). Ca^2+^ uptake is respiration-dependent, thus high rates of uptake can effectively lower the mitochondrial membrane potential (ΔΨ_m_) (reviewed in [15]). This phenomenon can be observed clearly in isolated mitochondria at supraphysiological concentrations of extramitochondrial Ca^2+^; however, under physiological conditions of Ca^2+^ uptake, a change in ΔΨ_m_ has not been detected in isolated cardiomyocytes using fluorescent indicators. Thus, Ca^2+^ uptake would not normally be expected to lower the membrane potential, at least for a sustained period, or to such an extent that it would inhibit ATP synthesis. Nonetheless, transient and small decreases in mitochondrial membrane potential may occur during cytosolic Ca^2+^ peaks in other systems, and can be observed in pancreatic β cells by following rhodamine-123 accumulation [16] or sensitive fluorescent ATP probes (Tarasov and Rutter, unpublished and see below).

The Ca^2+^ transport pathways of mitochondria are covered in more detail by other reviews in this issue. However, a brief discussion of their properties specifically with regard to energy production will be given here.

Early studies on isolated mitochondria characterised the MCU as a low affinity, high capacity transporter of Ca^2+^, whereas the mNCX had a much lower *V*_max_, saturating at [Ca^2+^]_m_ below 1 μM (reviewed in [17,15]). Thus, it was predicted that net Ca^2+^ influx would occur only when external [Ca^2+^] rose above about 500 nM, much higher than normal resting [Ca^2+^]_c_ of 100–200 nM [18]. Studies in living adult rat myocytes [Ca^2+^]_m_ confirmed this prediction, since [Ca^2+^]_m_ remained less than [Ca^2+^]_c_ until the latter rose above 500 nM [18]. However, the seminal paper by Rizzuto, Pozzan and colleagues in 1992, using aequorin targeted to mitochondria, revealed that these organelles were capable of taking up Ca^2+^ on a fast timescale, due to their proximity to intracellular Ca^2+^ stores [10] so they would effectively “see” much higher [Ca^2+^]_c_ than that of the bulk cytosol. In some cells and species [Ca^2+^]_m_ has been reported to change in response to physiological, submicromolar, changes in [Ca^2+^]_c_; but, in cardiomyocytes for example, the reflected changes in [Ca^2+^]_m_ were small, 10–20 nM [19].

Research into the mitochondrial Ca^2+^ transporters is now likely to undergo another step change, with the identification of proteins corresponding to both MCU and mNCX. Two papers published simultaneously in 2011 identified a Ca^2+^ uptake pathway in mitochondria, inhibited by ruthenium red: Baughman et al. [20] identified the MCU as a novel predicted transmembrane protein (CCD109A) that formed oligomers in the inner membrane, and interacted with their previously identified regulator protein, MICU1. Silencing MCU in cultured cells or in vivo in mouse liver abrogated Ca^2+^ uptake, but membrane potential and respiration remain intact. A point mutation removed the sensitivity to ruthenium red (RuR). The other paper [21] identified MCU as a 40 kD protein with 2 transmembrane regions. RNA silencing of MCU in HeLa cells reduced mitochondrial Ca^2+^ uptake, and purified MCU reconstituted in bilayers exhibited Ca^2+^ channel activity that was sensitive to RuR.

In 2010, Palty et al. [22] identified NCLX in mitochondria – this protein is a novel NCX, lying in an ancestral branch of the NCX superfamily. The protein occurred as 50, 70 and 100 kD forms in mitochondrial fractions of mouse heart and brain; the 100 kD form likely representing dimers. The authors demonstrated that mitochondrial Ca^2+^ efflux was enhanced upon over-expression of NCLX in HEK 293 cells and inhibited by CGP 371457, and that Ca^2+^ efflux was greatly reduced using an anti-NCLX siRNA.

## 4 Ca^2+^ sensitive mitochondrial enzymes

### 4.1 FAD-glycerol phosphate dehydrogenase (FAD-GPDH)

Physiological levels of [Ca^2+^]_c_ regulate FAD-GPDH (*K*_0.5_ ≃ 0.1 μM in rat liver mitochondrial extracts [23]), via a cytosol-facing EF-hand binding site for Ca^2+^ [24]. Increases in extramitochondrial Ca^2+^ lowered the *K*_M_ for DL-glycerolphosphate (*K*_0.5_ = 30–100 nM) in permeabilized INS1 β cells [25].

### 4.2 Pyruvate dehydrogenase phosphatase (PDHP)

The 50 MDa pyruvate dehydrogenase (PDH) multi-enzyme complex catalyses the irreversible reaction: pyruvate + CoA + NAD → acetyl-CoA + NADH_2_ + CO_2_, with the product, acetyl-CoA, then entering the citrate cycle or fatty acid synthesis. The complex comprises multiple copies of three enzymes: E1 (that decarboxylates pyruvate); E2 (forms acetyl-CoA) and E3 (reduces NAD^+^ to NADH).

The activity of the PDH complex is a rate-limiting step for glucose oxidation and is therefore reflected in the rate of respiration and ATP synthesis in mammalian tissues [26]. The regulation of the PDH complex activity is achieved via end-product inhibition and reversible phosphorylation by a PDH kinase, which inhibits the activity of E1 subunit via phosphorylation at three sites around S293 [27,28]. A Mg^2+^-dependent PDH phosphatase, in turn, dephosphorylates the E1 subunit.

The activity of the Ca^2+^-regulated phosphatase depends on the association of the catalytic subunit of the predominating isoform 1 of PHD phosphatase (PDHP1c) and the L2 domain of the E2 subunit of the PDH complex, which is potentiated by Ca^2+^ [29]. PDHP1c and the L2 domain of the E2 subunit lack Ca^2+^ binding motifs so the binding site for the cation is likely to be formed jointly by residues of both interacting proteins [30]. Ca^2+^ activates the phosphatase in heart mitochondrial extracts with *K*_0.5_ of ∼1 μM [31] and extramitochondrial Ca^2+^ stimulates phosphatase activity over the physiological range (0.1–1.0 μM), in rat heart [32] liver [33] and adipose tissue [34] mitochondria.

### 4.3 NAD^+^-isocitrate dehydrogenase (NAD-ICDH)

NAD-ICDH is a hetero-octamer consisting (in eukaryotes) of 2 × (2*α*,*β*,*γ*) subunits [35]. The enzyme binds ∼2Ca^2+^/octamer [7] but the binding site is as yet unknown. In the presence of ADP, Ca^2+^ decreased the *K*_*M*_ for isocitrate ∼8-fold [36]) with a *K*_0.5_ for Ca^2+^ of 1.2 μM in mitochondrial extracts [37] and 5–43 μM (increasing with ATP/ADP ratio), in toluene-permeabilised mitochondria [38]. ADP (or ATP) and Mg^2+^-isocitrate are required for Ca^2+^ binding [7]. The lower sensitivity of this enzyme to Ca^2+^ compared to other intramitochondrial dehydrogenases may extend the range over which Ca^2+^ is able to modulate oxidative metabolism by these organelles [8].

### 4.4 2-Oxoglutarate dehydrogenase (OGDH)

The OGDH complex, similarly to the PDH complex, consists of multiple copies of three subunits: E1 (α-ketoacid decarboxylase), E2 (dihydrolipoyl transacetylase) and E3 (dihydrolipoamide dehydrogenase) [39]. The activity of the complex is directly regulated by Ca^2+^, most likely via the E1 subunit [40] (*K*_0.5_ = 1–7 μM in mitochondrial extracts) [7]. Each enzyme complex binds ∼3.5Ca^2+^ ions, suggestive of a Ca^2+^ binding site at E1 dimer interfaces [7]. Ca^2+^ decreases the *K*_*M*_ for 2-oxoglutarate with little effect on *V*_max_ [41]. The *K*_0.5_ for extramitochondrial Ca^2+^ is ∼0.15 μM in intact heart mitochondria [37,42].

### 4.5 F_1_–F_O_ ATP synthase

The possibility that the rate of ATP production can be regulated independently from the respiration rate or mitochondrial membrane potential (Δψ_m_) has been demonstrated in intact mitochondria [43] and confirmed using control theory analysis [44]. Early in vitro [45] and in vivo [46] studies reported F_1_–F_O_ synthase as a potential target of this regulation. Although shown to bind Ca^2+^ directly [47], the F_1_–F_O_ ATP synthase is likely to be regulated via post-translational modifications and phosphorylation of the γ-subunit was sensitive to mitochondrial Ca^2+^ [48] with a *K*_0.5_ in the μM range. Recent work has identified a protein that binds to the F_1_–F_O_ synthase in Ca^2+^-dependent manner, resulting in an increased capacity for ATP production [49].

### 4.6 Other potential mitochondrial targets for Ca^2+^

*Cytochrome c oxidase* (COX) is allosterically inhibited by ATP/ADP (binding to the matrix domain of subunit IV). This inhibition is switched on by the cAMP-dependent phosphorylation of subunits II (and/or) III and Vb by protein kinase A (PKA), and results in an enhanced H+/e-stoichiometry and therefore more efficient energy transduction. Ca^2+^-dependent phosphatase reverses the cAMP-mediated effect (in vitro or in isolated mitochondria) [50]. cAMP is likely to stimulate the phosphorylation at Tyr304 of subunit I, which decreases the *V*_max_ and increases the *K*_*M*_. COX binds Ca^2+^ with a *K*_*d*_ of around 1 μM, competitively with Na^+^ [51].

*Malate–aspartate shuttle activity*. Ca^2+^ activates this shuttle in brain mitochondria (*K*_0.5_ = 0.3 μM) [52]. The aspartate–glutamate transporter has a cytosolic EF domain and is also regulated by [Ca^2+^]_c_, albeit in the supraphysiological range [53].

*Pyrophosphatase* activity in liver and heart mitochondria is inhibited by Ca^2+^, due to competition of CaPPi with MgPPi (*K*_0.5_ = 12 μM) [54]. The accumulation of PP_i_ is expected to result in an increase in mitochondrial volume and activation of the respiratory chain.

*The ATP-Mg*^*2+*^*/P*_*i*_ *transporter (SCaMC1)*. This transporter is stimulated by extramitochondrial Ca^2+^ and may regulated the total content of adenine nucleotides inside mitochondria, and hence alter Ca^2+^ buffering. SCaMC1 is overexpressed in some cancer cell lines, and this has been proposed to contribute to the ability of the cells to withstand Ca^2+^ loads that would otherwise trigger opening of the mitochondrial permeability transition pore [56].

Ca^2+^ reportedly potentiates fatty acid oxidation to β-hydroxybutyrate [55] though the underlying mechanisms are not understood.

## 5 Role of mitochondrial Ca^2+^ transport in specific cell types

### 5.1 Cardiac myocyte

**Fig. 1:**
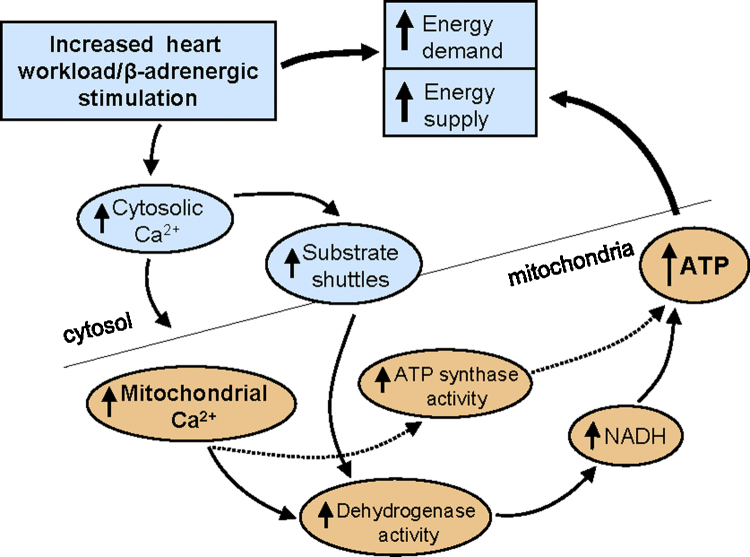
Role of Ca^2+^ uptake by mitochondria in the heart.

Fig. 1 presents a summary of the model proposed for mitochondrial Ca^2+^ uptake in the control of ATP synthesis in the heart.

Oxidative phosphorylation in mitochondria provides over 90% of ATP production in the heart [45]. The overall equation is given below, although estimates of the number of ATP molecules synthesised per molecule of NADH vary:

NADH + H^+^ + O_2_ + 2.5ADP + 2.5P_i_ → NAD^+^ + H_2_O + 2.5 ATP

ATP production can potentially be regulated by several different mechanisms, such as increases in NADH/NAD^+^ and ADP/ATP. It was originally proposed that changes in the ADP/ATP ratio were the main regulator of ATP synthesis [2], and this was easily demonstrated in isolated mitochondria. However, later experiments using isolated beating rat hearts found that high energy phosphate levels did not change under conditions that imposed a large increase in energy demand, e.g. increases in workload of the heart [57,58]. In fact utilisation of ATP under these conditions was exactly matched by an increase in ATP production. Changes in ATP are initially buffered by the phosphocreatine system but this only lasts for a few seconds, implying the existence of alternative mechanisms for rapidly increasing ATP synthesis.

This role was later found to be fulfilled by intramitochondrial Ca^2+^, following the discovery of the activation by Ca^2+^ of mitochondrial enzymes, as discussed above, namely the matrix dehydrogenases PDH [31], NAD-ICDH [37], OGDH [9], and possibly the ATP synthase [59]. The observations led Denton, McCormack and colleagues to propose that an increase in the supply of reducing equivalents in the form of NADH and FADH_2_ resulting from Ca^2+^ activation of the dehydrogenases would increase ATP production; thus suggesting a parallel activation model of stimulation of contraction and ATP synthesis by Ca^2+^ [60,61] (and see Fig. 1). The mitochondrial F_1_F_0_ATP synthase (ATPase) may also be stimulated by Ca^2+^ in this setting [43,47,62,63].

Whether Ca^2+^ transport into mitochondria occurs in the heart, and whether or not [Ca^2+^]_m_ changes on a beat-to-beat basis in this organ, has remained controversial for some time. Based on work in isolated mitochondria, the MCU and mNCX pathways could certainly not respond quickly enough to the very rapid (ms) changes in [Ca^2+^]_c_ that occur during excitation-contraction (EC) coupling. However, more recent work using isolated myocytes has suggested that mitochondrial Ca^2+^ transients do occur during EC coupling in neonatal and adult cardiac myocytes [64–66]. There is conflicting data on this in the literature since other studies reported that mitochondrial Ca^2+^ accumulation occurred over tens of seconds in cardiac myocytes [18,67,68]. Part of the conflict may be due to different species or stage of development (neonate versus adult), although why and how mitochondrial Ca^2+^ spiking occurs in some species but not others remains unknown. However, there is agreement that the main role of MCU and mNCX under physiological conditions is to translate or decode cytosolic Ca^2+^ signals in the mitochondria, so that Ca^2+^ activation of mitochondrial metabolism occurs.

In addition to the known proximity of mitochondria to the ER in a variety of cell types, there is now evidence for a direct physical coupling that persists during isolation of mitochondria (reviewed in [69]). This evidence was provided in heart by isolation of mitochondrial fractions with associated SR components that were highly resistant to purification [70]. These “SR appendices” were capable of transferring Ca^2+^ directly to mitochondria (measured with rhod-2 fluorescence) upon stimulation with caffeine (which activated the ryanodine receptor, thus triggering release of Ca^2+^ from the SR), and of increasing the NADH autofluorescence signal, suggesting activation of the dehydrogenases. This was the first direct evidence that Ca^2+^ transfer directly from the SR to mitochondria is capable of activating oxidative metabolism [70].

Activation of ATP synthesis in the heart by mitochondrial Ca^2+^. Parallel measurements of [Ca^2+^]_m_ and [Ca^2+^]_c_ in rat myocytes revealed that resting [Ca^2+^]_m_ was about 80 nM compared with resting [Ca^2+^]_c_ of 150 nM [18]. Whereas in mitochondria isolated from beating hearts the [Ca^2+^]_m_ was estimated to be slightly higher, about 170 nM [71]. Upon rapid stimulation in presence of an adrenergic agonist (myocytes), or increased workload (isolated hearts), the value of [Ca^2+^]_m_ increased to 500–1000 nM [18,71]. These values of [Ca^2+^]_m_ under physiological conditions are within the range for activation of the dehydrogenases (see above).

There is also evidence to suggest that extramitochondrial Ca^2+^ regulates substrate supply into mitochondria via the aspartate–glutamate carrier (aralar) and the malate–aspartate shuttle (see above). This newer mechanism has been proposed to act as a “gas pedal, supplying…substrate on demand” [72].

A problem in elucidating the relationship between [Ca^2+^]_m_ and [ATP] was that although ATP has been measured in whole hearts, and now in animals and humans using non-invasive ^31^P-NMR (reviewed in [73]), only relatively slow responses were measured, and so it could not be determined whether ATP was varying beat-to-beat, or during the time taken for mitochondria to accumulate sufficient Ca^2+^ to activate the dehydrogenases.

We directly measured ATP in mitochondria and cytosol, [ATP]_m_ and [ATP]_c_, respectively, using targeted luciferase [64], and [Ca^2+^]_m_ and [Ca^2+^]_c_ using targeted aequorin: in adult cardiac myocytes stimulated to contract at 2 Hz, there was no change in [ATP] in either compartment on addition of isoproterenol, despite an increase in contractile force and increases in both [Ca^2+^]_m_ and [Ca^2+^]_c_. This indicated that [ATP]_c_ was extremely well-buffered in myocytes, i.e. that the rate of ATP supply and/or synthesis can keep pace with that of ATP breakdown, even under conditions that imposed a large energy demand on the cell. This lack of change in [ATP] is in agreement of studies using whole hearts [74].

However, when we allowed cells to rest, then suddenly stimulated to contract in the presence of isoproterenol (“standing-start” experiments), [ATP]_m_ showed a significant transient drop (up to 22% in some cells) followed by recovery to higher than initial levels [64]. These changes likely reflect an initial activation of ATP-requiring processes in the cytosol, such as ion pumps and contractile proteins, which would cause a drop in [ATP]_m_ before a time-dependent activation of mitochondrial oxidative metabolism by Ca^2+^ stimulated ATP synthesis [75]. The changes in [ATP]_c_ were much smaller than those in the mitochondrial matrix, again suggesting that even when [ATP]_m_ does change, the cytosolic energy supply is very rapidly matched to the increased demand. The lag phase where [ATP]_m_ falls before recovering, exactly matches the time course for [Ca^2+^]_m_ to increase [64,76].

Similarly it has been generally observed that under conditions of adequate oxygen consumption, NAD(P)H levels remain constant in beating hearts and myocytes, again highlighting that ATP supply and demand are extremely well-coupled. The only time NAD(P)H was observed to decrease was in similar experiments to the “standing start” experiments described above, where there was an initial drop in NAD(P)H levels before recovery, again being slightly preceded by an increase in [Ca^2+^]_m_ [77].

Do ADP and Ca^2+^ have joint role in regulating ATP production in myocytes? Balaban [73] has argued that, given the importance of ensuring a rapid response system of ATP synthesis in the myocardium, more than one mechanism is likely to operate to coordinate ATP supply and demand. Whether [Ca^2+^]_m_ is absolutely required to maintain ATP levels during continuous or large increases in energy demand is not yet known and the idea of [Ca^2+^]_m_ being the only factor that is important physiologically in regulating ATP supply and demand is not universally accepted. Our own studies show that in rat cardiac myocytes when stimulation rate is increased from 0.2 Hz to 4 Hz, which presumably causes a large increase in ATP demand, there is very little change in [Ca^2+^]_m_ unless an adrenergic agonist is also present [67]; presumably this may increase the systolic [Ca^2+^]_c_ sufficiently that MCU is activated to increase [Ca^2+^]_m_.

One possibility is again that of the subcellular arrangement of mitochondria and proximity to other cell compartments: Saks and colleagues have put forward the idea that the subcellular architecture changes during contraction, causing a decrease in the apparent *K*_*M*_ of ADP for stimulating respiration, and removing diffusion limits of ADP that occur in resting cells (reviewed in [78]). This requires the mitochondria to be organised in structural units with ATP consuming processes in the cell, such as myofibrils, SR and sarcolemmal ATPases, so-called “intracellular energy units (ICEUs)” – where channelling of ADP occurs.

This group also argues that [Ca^2+^]_m_ may not be the sole factor, or possibly not a major factor, in regulating ATP supply and demand in the heart, since modelling studies have predicted that the increase in [Ca^2+^]_m_ in the heart would only be enough to stimulate respiration 2–3 fold, whereas increases of 10–20-fold are seen in vivo [43,79]. Therefore localised regulation of [ADP] in the ICEUs would present a method of stimulating ATP synthesis by mitochondria without changes in bulk [ADP] or [ATP] [80]. However, it is not clear how far these studies can be extrapolated to the physiological situation of rapidly beating cardiac myocytes, either cells or whole hearts that are continually shortening and re-lengthening. If the authors are correct, the theory also implies that control of respiration by ADP is operating on a millisecond timescale (in rat cardiac myocytes, for example). Alternatively, respiration could be sensitive to the time-averaged local [ADP], in a similar manner as we suggested above for rapid changes in [Ca^2+^]_m_.

*Implications for cardiac disease*. Pathologically, an increase in [Ca^2+^]_m_ has been associated with the transition from reversible to irreversible cell injury in ischaemia/reperfusion injury of the heart, and a major contributing factor to this injury is Ca^2+^-induced opening of the mitochondrial permeability transition pore [81,82]. A role for [Ca^2+^]_m_ in heart failure, and for the mitochondrial Ca^2+^ efflux pathway in the treatment of this condition has also been suggested [83].

Both MCU and mNCX have been proposed as possible targets for cardioprotective drugs (reviewed in [76]). However, protective effects of RuR have been found in ischaemia reperfusion injury ([84,85]), although it is not certain that the drug was acting on MCU since it is not specific and also affects SR Ca^2+^ flux [86]. Indeed we found, using a myocyte model of hypoxia/reoxygenation injury, that RuR was not protective [87], but that inhibiting the mNCX with clonazepam was. We concluded that the route of Ca^2+^ entry into mitochondria under hypoxic conditions is likely to be the mNCX rather than MCU [87,88].

The mNCX has also been considered a target for cardioprotection in heart failure. Recent work from O’Rourke's group has shown that dysregulation of Na^+^ homeostasis in heart failure may be a primary cause of mitochondrial dysfunction: in a guinea-pig model of heart failure (induced by aortic constriction), intracellular [Na^+^] was 16 mM compared with 5 mM in control cells [89]. Rapid pacing of the cells induced a decrease in NADH fluorescence, an indirect indicator of respiratory chain activity, whereas this was maintained in controls. CGP 37157, an inhibitor of mNCX, was able to prevent the decrease in NADH in the failing myocytes. It is thus likely to restore ATP levels although this has yet to be shown directly: earlier work showed that the mNCX is capable of regulating [Ca^2+^]_m_ and dehydrogenase activity since adding Na^+^ to isolated mitochondria shifts the activation curves for PDH and OGDH by Ca^2+^ to the right [32].

### 5.2 The pancreatic islet β-cell

Insulin secretion from pancreatic β-cells is prompted by increases in blood glucose over the physiological range (4–8 mM), thanks to the expression of high *K*_*M*_ glucose transporters (Glut2 in rodents) and glucokinase [90]. Defective β-cell glucose sensitivity [91,92] as well as a decrease in numbers of these fuel-sensing cells [93,94] result in hyperglycaemia and eventually type 2 diabetes.

**Fig. 2:**
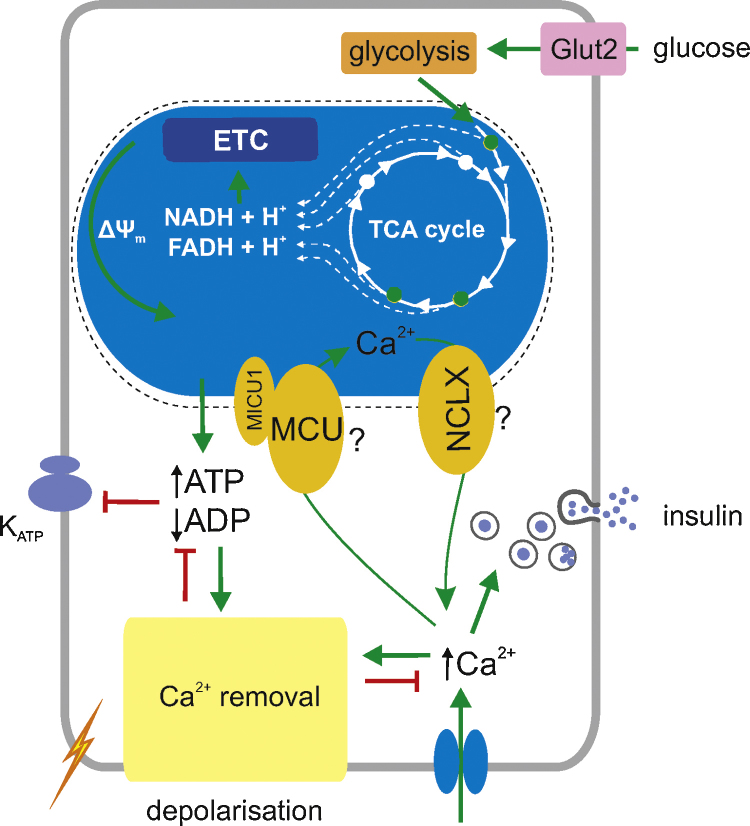
Potential role of Ca^2+^ uptake by mitochondria in the pancreatic β-cell. ETC, electron transport chain. See the text for further details of the Ca^2+^ sensitive intramitochondrial dehydrogenases.

The metabolic configuration of β-cells is adapted to favour the complete oxidation of glucose by mitochondria [95] through the suppression of genes involved in the production of lactate (LDHA and the plasma membrane monocarboxylate transporter, SLC16A1/MCT1) [96–99], and the expression at high levels of FAD-GPDH [24,25,100]. Consequently, increases in extracellular glucose are obligatorily linked to increased flux through the citrate cycle [100] and lead to clear elevations in cytosolic ATP/ADP ratio [101], which block ATP-sensitive K^+^ (K_ATP_) channels on the plasma membrane [102]. This triggers plasma membrane depolarisation, electrical activity and Ca^2+^ influx into the cytosol via voltage-gated Ca^2+^ channels [103]. The elevated [Ca^2+^]_c_ then triggers exocytosis of insulin granules [90,104] (Fig. 2). Other, more poorly defined “K_ATP_-independent” effects of glucose, possibly involving the inhibition of AMP-activated protein kinase [105,106], also contribute to “amplifying” effects of the sugar on secretion [107]. Changes in ATP/ADP ratio also regulate exocytosis directly [108], modulating the effects of cAMP [109,110].

In contrast to most tissues including the heart (see above), cytosolic ATP changes over a relatively wide range in β-cells [111] and this is likely to play a key-signalling role. Our early measurements of glucose-induced ATP dynamics in MIN6 and primary islet β cells [75,112,113], using the recombinant bioluminescent reporter firely luciferase (an approach whose sensitivity is relatively poor), suggested a monophasic elevation of ATP occurs in response to high glucose, although evidence for oscillations was also obtained [113,114]. Significant differences between the glucose-induced changes in the bulk cytosol and a sub-plasma membrane “microdomain”, as well as the mitochondrial matrix, were also demonstrated [75]. Importantly, blockade of Ca^2+^ influx with EGTA or through the inhibition of voltage-gated Ca^2+^ channels substantially inhibited glucose-induced ATP/ADP increases, implicating a role for Ca^2+^ influx in metabolic control [113].

**Fig. 3:**
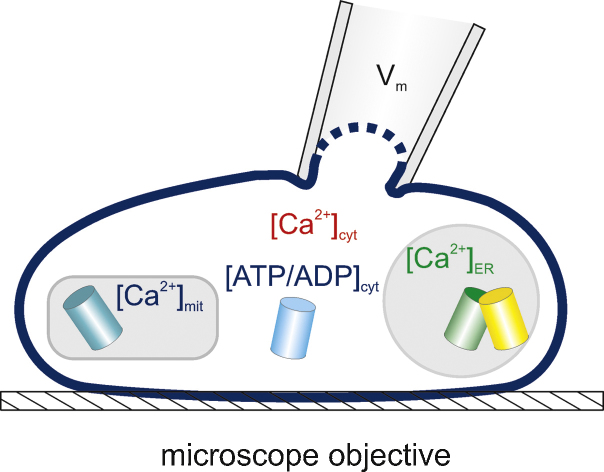
Principle of simultaneous patch-clamp recording and multiparameter imaging of compartmentalised Ca^2+^ and ATP/ADP in single cells.

**Fig. 4:**
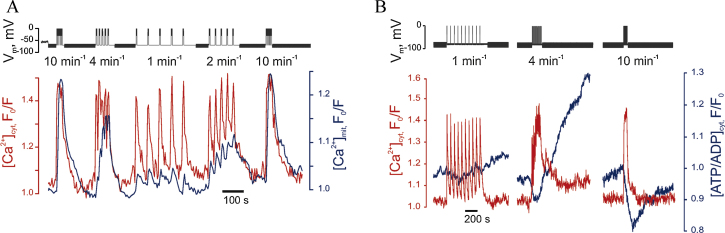
Mitochondrial Ca^2+^ (A) and [ATP/ADP]_cyt_ (B) increases respond to the frequency of electrical bursting and Ca^2+^ oscillations in a single primary mouse β-cell. Depolarisations of the plasma membrane were applied at different frequency from 10 min^−1^ to 1 min^−1^[115].

**Fig. 5:**
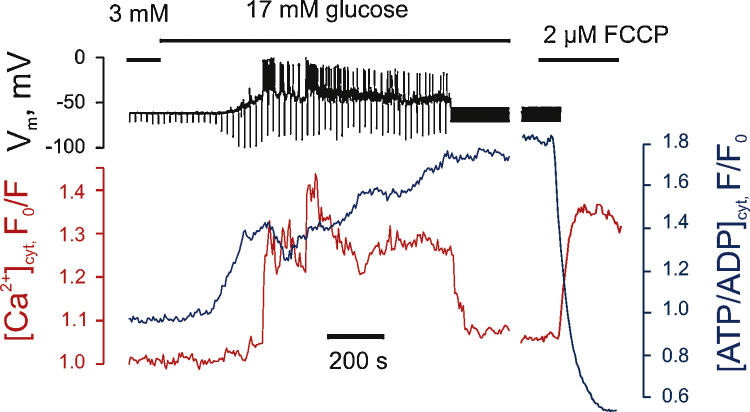
Multiphasic increases in [ATP/ADP]_c_ are prompted by high glucose in the β-cell. Recordings and image collection were as in Fig. 4; note the second phase of ATP/ADP increase ∼400 s after the increase in glucose [115].

In light of the above limitations, we have recently established a technique whereby [Ca^2+^]_m_ can be imaged in real time in single primary living β-cells using a mitochondrially targeted, adenovirally-delivered and GFP-based reporter (“Pericam”; 2mt8RP) simultaneously with [Ca^2+^]_c_ measured with the trappable small molecule, Fura Red (Fig. 3). Importantly, after the formation of a perforated patch, imaging of the two probes can be performed simultaneously with recordings or manipulation (at will) of plasma membrane potential. This approach demonstrates that [Ca^2+^]_m_ is unable to track fast oscillations of [Ca^2+^]_c_, imposed by single action potentials that have the duration of 50–200 ms in β-cells [115]. However, [Ca^2+^]_m_ does follow slow changes in [Ca^2+^]_c_ similar to those associated with slow electrical bursting [115]. This “accumulation” mechanism makes [Ca^2+^]_m_ highly sensitive to the frequency of Ca^2+^ oscillations [115] (Fig. 4), such that low frequency spiking barely affects [Ca^2+^]_m_. This “Frequency-Amplitude” demodulation may thus allow filtering of low level stimulation (for example at low glucose concentrations) whilst amplifying the response as glucose levels rise – thus contributing to the well-known highly sigmoidal response of insulin secretion to blood glucose levels [95]. This positive feedback may play an important role in coordinating insulin secretion in pancreatic islets where β-cells are electrically coupled [116]. Using the same approach to combine imaging of [Ca^2+^]_c_ and [ATP/ADP]_c_, using the genetically encoded fluorescent sensor Perceval [117], we have been able to demonstrate a biphasic increase in [ATP/ADP]_c_ in response to high glucose, with Ca^2+^ entry into cytosol being the key factor for the second phase [115] (Fig. 5).

These observations are consistent with a model for the β-cell in which elevation of [Ca^2+^]_c_ leads firstly to the activation of a number of processes (e.g. Ca^2+^ pumping [118,119]) that elevate ATP consumption [101,113,115]. This is then matched by a Ca^2+^-dependent potentiation of mitochondrial metabolism [115,120]. The existence of these two phases thus generates a biphasic increase in [ATP/ADP]_c_. (Fig. 5). Whilst the role in stimulus-secretion coupling of the second phase is unclear, an intriguing possibility is that it is involved in the mobilisation of secretory granules from a “reserve pool” in the cell [95].

Consistent with these findings, silencing of MCU [115], or deployment of an intramitochondrial Ca^2+^ buffer [120], blocks both mitochondrial Ca^2+^ uptake and the second phase of ATP/ADP increase. Interestingly, glucolipotoxic conditions mimicking the diabetic milieu and known to affect mitochondrial integrity [121], suppress [Ca^2+^]_m_ changes and selectively reduce the second phase of [ATP/ADP]_c_ increase [115]. Defective mitochondrial Ca^2+^ handling may thus play a part in the defects in insulin secretion in type 2 diabetes.

## 6 Conclusions

There is no doubt that [Ca^2+^]_m_ plays an important role in both myocardial energy production and in intracellular Ca^2+^ signalling in many other systems including the pancreatic β-cell. Future challenges will involve further dissection of the molecular mechanisms involved (including identification of the Ca^2+^ binding sites on the mitochondrial dehydrogenases) and exploration of the potential therapeutic use of new information flowing from the identification of the molecular players in Ca^2+^ transport.

## Conflict of interest

None of the authors declares any conflict of interest.
